# Understanding employee creativity from the perspectives of grit, work engagement, person organization fit, and feedback

**DOI:** 10.3389/fpsyg.2022.1012315

**Published:** 2023-01-27

**Authors:** Miapeh Kous Gonlepa, Sana Dilawar, Tunde Simeon Amosun

**Affiliations:** ^1^School of Public Affairs, University of Science and Technology of China, Hefei, China; ^2^Department of Sci-Tech Communication and Policy, School of Humanities and Social Sciences, University of Science and Technology of China, Hefei, China

**Keywords:** grit, work engagement, person-organization fit, feedback, creativity

## Abstract

**Purpose:**

Drawing on the growing emphasis in the literature on the importance of creativity in the workplace, the present study examines the effect of personal and organizational level factors that influences employee creativity. Precisely, we examine how grit, work engagement, person-organization fit, and feedback influence creativity in the workplace.

**Design/methodology/approach:**

We sampled data from 422 research assistants who are professional workers at top-notch Universities in China. They were recruited to participate in the survey through an online medium known as WeChat. We empirically tested the effect of grit, work engagement, person-organization fit, and feedback on employee creativity. These hypotheses were supported by confirmatory and exploratory factor analysis, and path analysis.

**Findings:**

The results show that work engagement strengthens the relationship between grit and creativity. In addition, the results proved that person-organization fit positively moderates the link between grit and work engagement. Feedback also had a positive mediating effect on the link between work engagement and creativity.

**Practical implications:**

Based on the appropriateness of an individual work environment, a gritty person will likely become engaged and creative with task execution. Consequently, a person’s organizational fit will strongly interact with grit, work engagement, and creativity.

**Originality/value:**

We shed light on the blended value of personal and organizational-level factors that positively affect creativity in the workplace. Specifically, grit being both a personal and organizational factor influences employee creativity *via* work engagement. This research explored the effect of work engagement as a mechanism that serves as a motivational feature enhancing creativity. We also simultaneously identify the moderation conditions of person-organization fit and feedback. The theoretical and practical implications of the findings are discussed in detail. The study makes a theoretical contribution through its assessment of the impact of grit on employee creativity. The trait activation theory portrays how grit can be expressed through feedback and person-organization fit. In terms of practice, grit can be an important consideration in hiring decisions, and feedback should be given to make the workplace more creative.

## Introduction

In today’s knowledge-based economy and dynamic business environment, different organizations are placing a high focus on employee creativity to gain a competitive and sustainable advantage (Mubarak and Noor, 2018; Bakker et al., 2020). Creativity has been identified as a key element in facilitating social and economic reform (Zhou and Shalley, 2011) while improving individual performance (Amabile, 1998). Research has pointed out that creativity is the process by which people develop new and useful ideas or solutions to problems. Several pieces of studies indicated that employee creativity relates to employee high growth needs, strength, and employee learning orientation (Gong et al., 2009; Shalley et al., 2009) as well as ensures organizations remain competitive, productive, and relevant in the global market sphere (Yoshida et al., 2014; Gu et al., 2015; Ouakouak and Ouedraogo, 2017). This elucidates why many organizations strive so hard to ensure employees are well-motivated to relish the outcomes of employees’ creativity (Liu et al., 2012; Gu et al., 2015). Extant studies examined the dynamics that revolve around creativity and identified personality traits and abilities significantly related to individuals’ creativity (Li et al., 2020). For instance, studies showed that extroverts show a high sense of creativity (Woodman et al., 1993).

In addition, some behavioral processes such as social learning (Tims and Parker, 2020); networking (Baer, 2010); and help/feedback-seeking attitude (De Stobbeleir et al., 2011; Mueller and Kamdar, 2011) strongly predict creativity. Based on these findings, scholars recommended the need for suitable job crafting for employee creative performance (Tian et al., 2021). Although this is necessary, it is equally vital to note that creativity does not solely depend on who is creative and how an organization’s working environment engenders substantial employee creative outcomes but also has to do with what employees themselves must do to contribute to their creativity. In this light, further research is required to aid employee self-realization of inherent traits and the mechanisms that expedite such traits for high levels of employee creativity and outcomes. Much is unknown about the role of grit on employee creativity in these prior investigations as a result, exploring vital factors such as person-organization fit and feedback can stimulate employee creativity and enhance organizational performance.

Based on trait activation theory (TAT), scholars devoted considerable attention to the special role of grit in organizational performance. Grit, a new construct in the field of behavioral science (Jordan et al., 2019b) is a highly significant predictor of an employee and organization’s success (Mueller et al., 2017; Dugan et al., 2019) and highlights “passion and perseverance for achieving long-term objectives” (Duckworth et al., 2007; Crede, 2018). Past studies established that higher levels of grit effectively relate to employee creativity and positive attitude toward work engagement (Chandrawaty and Widodo, 2020). Scholars concluded that when employees demonstrate high levels of passion and tenacity, it can positively alter the value that such employees attach to such work as well as kindle the employees’ creative (Suzuki et al., 2015; Chandrawaty and Widodo, 2020; Nisar et al., 2020).

A review of the literature emphasized feedback mechanisms accounting for grit’s developmental properties over time (Jordan et al., 2019b). Hartmann and Rutherford (2015) asserted that feedback from supervisors or colleagues promotes employees’ grit. Such a phenomenon indicates that employees can be creative in a positive work atmosphere. However, to the best of the authors’ knowledge, no study has examined the relationship between grit and creativity. This study aims at filling this gap. Given the insights from the TAT, this study also aims to incorporate the mediating role of work engagement and the moderating role of person-organization fit and feedback in the interplay between employee grit and creativity.

Drawing on the aforementioned gaps and arguments, this study makes multiple contributions. Theoretically, we contribute to organizational behavior literature by identifying grit, work engagement, person-organization fit, and feedback as determinants of employee creativity. Similarly, we add to TAT’s highlights on the effect of personality variables in the workplace (Tett and Burnett, 2003). This study shows that although grit positively relates to employee creativity it also does so *via* other imperative mechanisms. Precisely, grit affects creativity *via* the mediating effect of work engagement. In addition, we show the moderating effects of person-organization fit and feedback in these relationships. Empirically, there has been no empirical research into the effects of grit, work engagement, person-organization fit, and feedback on creativity at the workplace, specifically, research assistants’ creativity in Chinese universities. Consequently, this research has implications for the conditions that underpin the effect of these determinants in organizational behavior research. This insight could be replicated in similar contexts.

The remaining sections of this study are classified as follows. The literature review and theoretical background are presented. Thereafter, sections on methodology, results, discussion, and conclusion are included.

## Literature review

### Theoretical background

Personality traits are dominant conceptions in psychology and have been characterized in numerous ways (Phares and Chaplin, 1997). Presently, they are conceived to be intra-individual consistencies and inter-individual uniqueness in propensities to behave in identifiable ways in light of situational demands (Tett and Guterman, 2000). This indicates that the concept of trait should mainly encompass the interpretations regarding an individual’s behavior patterns and how the individual’s grit influences creativity. The TAT has provided insight into how personality is related to performance in the workplace as a response to trait-relevant cues (Tett and Burnett, 2003). Tett and Guterman (2000) argued that traits are a person’s latent potential and relevant situational cues can trigger trait expressions (i.e., behaviors). These situational cues may stem from an organization, social, and/or task cues. These cues can activate personality traits (i.e., grit) that are task-related cues (i.e., engaging in work activities) and organizational expectations that the organization values (i.e., employee creativity/performance). Although trait expression is a fundamental part of human nature, TAT emphasizes that the impact of a trait depends on the work situational cues provided (Tett et al., 2021) which can also be expressed in a person’s work behavior.

The response to situations is also an important factor for individual behavior in the workplace (Day and Silverman, 1989). The basic premise of trait activation is that the degree to which a trait is likely to drive behavior is a function of the extent to which the situation provides an opportunity for or creates a necessity for the trait (Tett and Burnett, 2003). For instance, the interaction of trait-related situational activators is a stimulus for one personality trait to manifest itself into conduct (Tett and Guterman, 2000). The importance of a trait and its context should be aligned such that the individual possesses the trait that allows them to respond effectively to the situation’s indications (Luria et al., 2019). Accordingly, scholars emphasized grit as individual behavior under three categorizations including consistency, perseverance, and interest (Duckworth et al., 2007). Taken together with recent endorsements, TAT gives insight into how personality is related to work engagement (Scharp et al., 2019).

Work engagement is defined as a positive affective-motivational state of fulfillment marked by vigor, dedication, and absorption (Demerouti et al., 2001). In this regard, scholars identified employees’ high levels of energy and enthusiasm (May et al., 2004; Macey and Schneider, 2008) as variables that affect work engagement. Other studies found that work engagement varies per individual due to work surroundings, personal characteristics, and behavioral strategies (Bakker and Xanthopoulou, 2013). Thus, work engagement leads to multiple positive-performance outcomes such as higher job satisfaction, lower intent to leave, and higher organizational commitment (Bakker and Leiter, 2010). Along these lines of emphasis, some scholars for instance, Zhang and Bartol (2010) endorsed the positive relationship between grit personality and engagement as well as work engagement and creativity in a work setting. Nonetheless, they concluded that the relationship between grit and creativity is not direct. This necessitates further exploration to answer the earlier call for empirical studies on the influencing mechanisms in the link between grit and employee creativity (Shalley et al., 2004).

Different perspectives showed a positive impact on employee creativity. First, from the standpoint of feedback (appropriate response), scholars noted that the TAT is appropriate to explain variance in employee creativity (Amabile and Pillemer, 2012) in different organizational contexts (Shalley et al., 2004; Zhang et al., 2011). Prior studies, for instance, Harding (2010) examined how creativity is related to leadership (appropriate responses) and work engagement. The study discovered that entrepreneurial leadership is crucial in promoting employee and team creativity by exhibiting creativity-favoring behaviors that are specifically tailored to workplace creative pursuits. Second, considerable evidence showed the predictive effect of person-organization fit (POF) and work engagement on employees’ creativity (Harding, 2010; Rich et al., 2010; Sarac et al., 2014). Yet, less research has used TAT to investigate how person-organization fit interacts with grit, work engagement, and feedback to promote creativity in the university setting.

### Conceptual framework and hypotheses development

**Figure 1:**
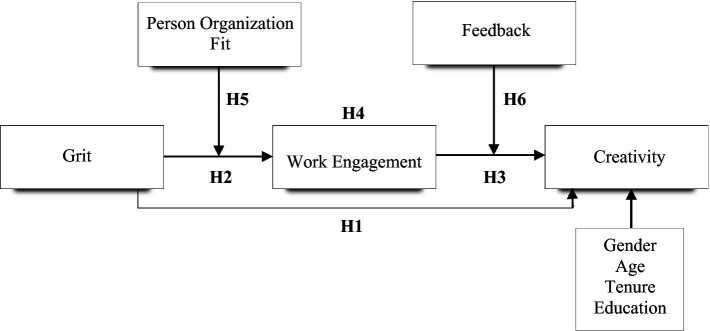
Conceptual model.

First, this study’s conceptual framework examines the potential effects of grit and work engagement on employee creativity from the perspective of TAT. At this stage, we also analyze the mediating effect of work engagement in the link between grit and employee creativity. Second, we examine two other mechanisms (feedback, and person-organization fit) based on the proposition that both factors interact with grit and work engagement (moderating effects) to influence employee creativity. The research model as shown in Figure 1 details the direct, mediation, and moderation factors as well as the hypotheses.

### Grit and employee creativity

Creativity plays a tremendous role in the progress of human endeavors as it relates to originality and novelty. Past studies argued that creativity is needed in almost every job or work (Shalley et al., 2000; Unsworth, 2001). Over the years, a lot of work has been conducted in the area of creativity, accounting for about 9,000 published articles (Runco et al., 1998). Creativity has been conceptualized in many different ways, however, the most widely recognized definition describes creativity as the rubrics involved in producing, conceptualizing, or developing new, and important ideas by an individual or group of individuals working in harmony (Shalley, 1991; Amabile, 1998). Employee creativity, therefore, describes the ability of an employee to generate new and useful ideas which are of crucial value to achieve effectiveness, innovation, and significant breakthroughs (Shalley et al., 2009; Liu et al., 2012; Yoshida et al., 2014). Extant studies also documented some factors that precede employee creativity. For instance, perseverance and passion are important predictors of creativity (Helson et al., 1995; Csikszentmihalyi, 1996). Moreover, both perseverance and passion are key subscales of the recent conceptualization of grit. Creative individuals show a high sense of perseverance. In the study conducted by Wilson (1990), through the process of observation, interviews, and psychological tests, it was revealed that poets persist or persevere in their writings even when they are confronted with a myriad of difficulties and challenges. Although the ability to persist or persevere is not only restricted to poets alone, it also actually depends on the domain in focus. In the field of education, women who have shown a high sense of occupational creativity 30 years after college are said to be those who displayed a high level of persistence or perseverance while in college despite the adversity (Helson et al., 1995). Perseverance has also been revealed to exert a strong and significant influence on the scientific creativity of laureates (Adelson, 2003). This implies that individuals need to be perseverant in the face of challenges to be creative. Similarly, creative individuals are passionate about any work they do (Csikszentmihalyi, 1988; Fisher and Amabile, 2008). Thus, passion influences employees’ creativity within a work setting (Liu et al., 2011). Earlier emphasis showed that passion affects the creativity of students majoring in performing arts (Vallerand et al., 2007). As a result of the various insights gathered from the review of the literature, we assume that grit which encompasses a blend of perseverance and passion can predict employee creativity. It is on this basis that the next hypothesis is developed:

*H1*: Grit is positively related to employees’ creativity.

### Employee’s grit and work engagement

Grit is defined as “perseverance and passion for long-term goals” (Bernardy and Antoni, 2021). Grit has been identified to have a strong relationship with positive outcomes such as an individual’s impulse for more education and higher performance at work (Duckworth et al., 2007); effective teaching behaviors (Duckworth and Quinn, 2009); lower burnout in surgical residents (Salles et al., 2014); increased hardiness of US Military Academy Cadets (Maddi et al., 2012); and increased level of soldier’s retention as well as sales workers (Eskreis-Winkler et al., 2014). Researchers also established that employees with a significant level of grit tend to go the extra mile to ensure that they get their jobs effectively done. We have, therefore, within the context of this study, conceptualized grit as an employee’s “perseverance and passion for long-term goals” with an organization setup.

In the literature, the conceptual disparities between grit and some of its related constructs have been established (Schimschal et al., 2021). A very common case in point is self-control and grit. The two concepts have been said to be success determinants regardless of the difficulties attached, however, self-control constitutes the qualities that an individual possesses in the process of effectively and efficiently reaching a resolution between two sets of unprompted actions. On the other hand, grit typifies the determined resolution toward long-term goals (Duckworth and Gross, 2014). In addition, conscientiousness is also associated with grit (Duckworth et al., 2007); however, grit highlights the discrepancy in the gradual increase of results (Duckworth et al., 2007), thereby implying a distinctive “construct domain.” The positive correlation between grit and hardiness is also reflected in Maddi et al. (2012)‘s definition of hardiness as a pattern of attitudes and skills that provides the existential form of courage and motivation needed for learning from stressful circumstances, to determine what will be the most effective performance. The above-cited examples have shown grit as a unique construct despite its relations with other related constructs.

Work engagement refers to the desire, zeal, and energy that an individual has for his or her work. This panoply of feelings can be grouped into three main dimensions, which include vigor, dedication, and absorption (Schaufeli and Bakker, 2004). Scholars further described work engagement as the feelings or action that exhibits strong positivity and fulfillment predicated on vigor, dedication, and absorption in a particular work situation. These three dimensions of work engagement according to scholars can be explained as follows: vigor represents the high degree of mental resilience and energy in the process of working, the readiness or disposition to exert one’s effort in the work, and the persistence when challenges and difficulties beckon. Dedication relates to a sense of significance, excitement, inspiration, pride, and challenge. Absorption is defined as being fully concentrated and happily engrossed in one’s task, such that time goes swiftly and it is difficult to disengage oneself from work.

Past empirical evidence revealed a positive relationship between grit and employee engagement in the workplace. Duckworth et al. (2007) conducted a study using US Military Cadets as its sample. The study showed that the grit component of the cadets was a significant predictor of their work engagement, subsequent performance, and retention in their military training. This goes to show that grit has the potential of stimulating the spirit of work engagement in individuals and has the proclivity to influence good performance in the workplace. In addition, other studies explored the association between employees’ grit, work engagement, and performance. One such study was conducted by Kim and Lee (2022). The findings showed that employees’ grit plays a positive imperative role in influencing employees’ quality of work life and in a similar manner affects their quality of life. It can be deduced from these findings that an employee who has higher grit is very likely to rate higher on work engagement level as a result of the positive effect that grit exerts on their quality of work-life and quality of life. The research of Von Culin et al. (2014) asserted that the discrepancies in terms of orientation as it relates to happiness might produce some sort of individual disparity in grit. In other words, grit represents a medium-sized relationship in connection with engagement, “*small-to-medium*” correlation with a predilection towards substance, and “*small-to-medium* (inverse)” relationship with a mindset directed at pleasure. Based on this study, Suzuki et al. (2015) indicated that individuals with a sense of happiness when engaging in work activities were more likely to attain success. Concisely, Suzuki et al. (2015) revealed that employee grit exerts a significant and positive influence on work engagement. Similarly, the study showed that when employees possess grit they are more likely to engage with their work totally compared to those without grit. Hence, it could be presumed that the grittier an employee, the potentially higher the influence, and subsequently, the work engagement.

As a result of the above-mentioned discussion, this study fairly assumes that an employee with a high level of grit will probably rate higher on the work engagement scale. The following hypothesis is therefore advanced:

*H2*: Grit is positively related to employees’ work engagement.

### Work engagement and employee creativity

Work engagement within an organization has remained imperative for the upward positive progress of an organization, agency, or company. It is also very essential when it comes to stimulating smooth working attitudes within an organization which could eventually influence organizational success. The study by Bakker et al. (2020) argued that employees who are diligently engaged with their respective work are very probable to show a high level of improvement in their creative performance in the workplace. Corroborating this assertion is the study of Asif et al. (2019) which posited that employees’ creativity, in an ethical leadership milieu is positively impacted in the workplace where employees are properly engaged with their work. This establishes the fact that there are empirical connections between the work engagement of employees and employees’ creativity. A positive link between the work engagement of school principals and their creativity has also been established (Bakker and Xanthopoulou, 2013). Based on the above arguments, we hypothesize that:

*H3*: Work engagement is positively related to employees’ creativity.

### The mediating role of work engagement

This study incorporates into the research framework the mediating role of employees’ work engagement in the relationship between employees’ grit and creativity in the workplace. Accordingly, we seek to extend the existing bodies of research on work engagement and creativity. Numerous studies explored the mediating role of employees’ work engagement in different contexts (Sulea et al., 2012; Yalabik et al., 2013; Gupta and Shaheen, 2017; Chaudhary and Akhouri, 2018; Coetzee and van Dyk, 2018). A conspicuous inference drawn from these studies pointed to the effectiveness of work engagement as a mediator between constructs that are related to different organizational activities, practices, and settings. In the study of Schaufeli and Bakker (2004) the association between job resources and turnover intentions was found to be mediated by engagement. In another study by Richardsen et al. (2006) work engagement was identified to partially mediate how individual features, JD-R (job demands-resources) affect organizational commitment and self-efficacy. In the same vein, work engagement fully mediates the association between job resources (variety, control, and feedback) and behaviors that are proactive (Salanova and Schaufeli, 2008). Some past studies also identified a positive relationship between employees’ grit and work engagement (Von Culin et al., 2014; Suzuki et al., 2015; Kim and Lee, 2022). Similarly, other previous studies affirmed the link between work engagement and employees’ creativity in the workplace (Bakker and Xanthopoulou, 2013; Asif et al., 2019; Bakker et al., 2020). Thus, we ask that apart from the foregrounded interplays, that is, between employees’ grit and work engagement; work engagement, and employees’ creativity, could there be any mediating influence that employees’ engagement exerts in the entire relationship? The quest to answer this question triggers the idea of integrating work engagement as a mediator. This results in developing the hypothesis below:

*H4*: Work engagement mediates the relationship between grit and employees’ creativity.

### The moderating role of person-organization fit and feedback-seeking

Person organization fit theory (POF) delineates the consistency or conformity that exists between the interests, values, aims, and objectives of the organization and those of the employees (Amah and Ahiauzu, 2014). In other words, “P-E” fit explains the alignment and congruence between the employees and their organization’s critical mission, vision, values, or objectives (Sarac et al., 2014). According to the study by Edwards (2008), it was asserted that employees’ attitude or behavior in the workplace is not only predicted by their personal features or the environment where the organization is situated but also by the relationship between these highlighted predictive factors. Past research established the fact that in a situation whereby there is a strong person-organization fit both the employees and organization tend to benefit significantly as well as achieve a productive uptick. Also, more commitment to organizational goals or objectives, increased satisfaction, and turnover diminishment are other noticeable benefits (Tepeci and Bartlett, 2002).

The associative relationship between employees’ grit and employees’ work engagement deserves to be explored further along the lines of moderating mechanisms like person-organization fit. An employee with high grit disposition tends to work harder with determination toward work engagement within the organization. The results of the study by Vogelsang (2018) showed a significant relationship between person-organization fit, grit, and task performance. Task performance in this instance is a behavioral outcome similar to work engagement that every organization invariably aims to evaluate at one point or the other. This lends credence to the assertion that a good person-organization fit may stimulate employees into leveraging their grit characteristics to strengthen their desire, zeal, and energy for work. In other words, it is not improbable that an employee with strong grit features combined with a good person-organization fit will engage more effectively and efficiently with the work and vice versa. This notion leads to the next hypothesis which highlights person-organization fit as a moderating construct.

*H5*: Person organization fit moderates the relationship between grit and work engagement.

Many scholars defined the concept of feedback in different ways as it relates to different contexts. However, for this study, the definition of feedback within the context of organizations becomes appropriate. In this sense, Ashford and Cummings (1983) explained feedback as “a subset of information available to individuals in their work environment.” Feedback is information that denotes how well individuals are meeting various goals. In the interpersonal realm, feedback involves information about how an individual’s behaviors are perceived and evaluated by relevant others. Research focusing on feedback within an organization is rapidly becoming an important area of interest. This is because, gifted or talented employees are seen as “the most valuable asset and the key to organizational success” (Whelan et al., 2010; van der Rijt et al., 2012). Feedback helps in stimulating employee learning and steady development that can engender viable performance in individuals (Maurer et al., 2003; Ericsson, 2009; Linderbaum and Levy, 2010; Salas et al., 2010). Other scholars also found that feedback plays a vital role in organizational competitiveness (Maurer, 2001), organizational effectiveness, and other work outcomes (Kluger and DeNisi, 1996). Feedback enhances work performance (Becker and Klimoski, 1989), affects organizational commitment (Norris-Watts and Levy, 2004), job motivation (Gagne and Deci, 2005), and creativity (Zhou, 1998). Findings in the study of Gabriel et al. (2014) revealed that feedback is strongly and positively related to the dimensions of employee psychological empowerment such as; meaning, competence, and self-determination. In other words, the feedback environment fostered by supervisors can increase employee empowerment. In another study by Anseel et al. (2011), feedback type was found to be an important moderator between achievement goals and task performance. It is therefore against the backdrop of extant literature review that this study integrates the moderating role of feedback in the relationship between employee work engagement and employee creativity. It is believed that feedback can strengthen how well an employee engages with work within a work organization. Similarly, past studies also identified feedback as highly significant in improving organizational performance, creativity, and innovation (De Stobbeleir et al., 2011). This study, therefore, comes up with the hypothesis below, situating feedback as a mediator:

*H6*: Feedback moderates the relationship between work engagement and employees’ creativity.

## Materials and methods

### Participants and procedures

Participants were Master’s and Ph.D. research assistants from top-notch universities in China and were engaged *via* WeChat. WeChat is the most extensive social media application in China. The research assistants meet the criteria of being knowledge workers because they are experienced, creative, and have high degrees of expertise in their field of specialization. Therefore, we believe that these participants are highly involved with creative engagement in their workplace.

A total of 450 respondents were expected to participate, however only 422 participated. From these, 21 were excluded during the screening stage due to incomplete information. The Kaiser-Meyer-Olkin Measure (KMO) approach was used to measure the 401 sample’s adequacy or strength (Kaiser, 1974). The resulting value of 0.814 exceeded the criteria of >0.50, showing that the sample size is adequate for this study’s estimations. The final sample of 401 consisted of 102 females (25.4%) and 299 males (74.6%). The majority of the participants were not more than age 29 (54.6%). Participants’ work experience was between 1 and 5 years (54.1%) (Table 1).

**Table 1:** Sample demographics statistics, *N* = 401.

Variable	Category	Frequency	Percent
Gender	Male	299	74.6
	Female	102	25.4
Age	18–29 years	219	54.6
	30–39 years	124	30.9
	40–49 Years	39	9.7
	50 years and above	19	4.7
Work experience	≤ a year	46	11.5
	1-5 years	217	54.1
	6-10 years	78	19.5
	≥ 10 years	60	15.0
Education	Masters	203	50.6
	Ph.D.	198	49.4

### Measures

All scale items were validated English scales from extant literature and were translated from English into Chinese and then translated back into English to confirm their contextual meaning was maintained (Brislin, 1980). We used a seven-point Likert scale (from 1 = strongly disagree to 7 = strongly agree) to measure each item.

### Grit

Participants completed the translated Chinese version of the short grit scale. The eight items on the short grit scale (Grit-S) developed by Duckworth and Quinn (2009) were used to assess participants’ grit-passion and perseverance for a long-term goal. The Grit-S comprised two subscales: perseverance and consistency of interest. The Grit-S is a simplified version of the original grit scale (Grit-O; Duckworth et al., 2007). Four items assessed the consistency of interest (e.g., “I often set a goal but later choose to pursue a different one”) and four items assessed the perseverance of effort (e.g., “I finish whatever I begin”), In this study, the Cronbach alpha for the scale was 0.786.

### Work engagement

We adopted the five scales from Utrecht Work Engagement Scale (UWES) from Schaufeli and Bakker (2004) to measure the extent to which employees are motivated to engage in their work. The five-scale was developed by Bledow et al. (2011). The affective shift model of work engagement assessed the three dimensions of work engagement—vigor, dedication, and absorption. Respondents were asked to indicate their agreement with items such as: (1) I feel strong and vigorous in my work; and (2) At my work, I feel bursting with energy. The Cronbach alpha for the scale was 0.958.

### Creativity

Creativity was assessed with four items which were adapted from the scale developed by Farmer et al. (2003). Respondents were asked to report their level of creativity by indicating their agreement with items such as: (1) I try new ideas or methods first; (2) and I seek new ideas and ways to solve problems. This scale was developed for the Chinese context to reveal the Chinese view of employee creativity. The internal consistency of the scale, measured with Cronbach’s alpha coefficient, was 0.935.

### Performance feedback

To assess the quality of the self-initiated feedback from the supervisor and coworker, we used one of the scales of widely used feedback environment scales from Steelman et al. (2004) that measures the feedback quality of the supervisor and coworker. The scale consists of 10 items, 5 items measured the self-initiated feedback quality from the supervisors, and 5 items measured the self-initiated feedback quality from the coworker. Thus, self-initiated feedback quality is associated with the informational value of the feedback message. It also describes the perceived consistency and usefulness of self-initiated feedback. These items focused on the quality of the feedback employees themselves have initiated from their supervisors and colleagues. Sample items are: (1) My supervisor provides me valuable feedback concerning the performance of my tasks; and (2) my coworkers’ performance feedback is beneficial. The Cronbach’s alpha for performance feedback was 0.953.

### Person-organization fit

We followed the definition of Kristof (1996). In her integrative review, she defined P-O fit as the compatibility between people and organizations that occurs when at least one entity offers what the other needs or shares similar essential characteristics, or both. Therefore, we evaluated P-O fit using three items from Cable and DeRue (2002). Cable and DeRue (2002) measured employees’ perceptions of P-O fit as a core work value by which employees judge their P-O fit by being drawn to organizations that exhibit characteristics similar to their own, and organizations, in turn, tend to choose individuals who are most similar to the organization. This scale has been validated in organizational behavior research (Cable and DeRue, 2002; Edwards, 2008). Sample items include: (1) The things I value in life are extremely similar to things my university’s values; and (2) My personal values align with my university’s values and culture. The Cronbach’s alpha for the scale was 0.784.

### Control variables

Age, gender, work experience, and education were chosen as control variables because they accurately reflect the respondents’ compositional situation. For the inclusion of these controls, we followed the advice of previous researchers (Adewale and Mustapha, 2015; Allena-Ozolina and Bazbauers, 2017). According to their hypothesis, the aforementioned controls will have a positive significant impact on human behavior.

### Data analysis

This section reports the data analytical strategy adopted to validate the proposed relationships of this study. The estimations were conducted using the Statistical Package for Social Sciences (SPSS) version 22 and Analysis of Moment Structures (AMOS) 24 software.

## Results

### Common method bias and multicollinearity analysis

To rule out the likelihood of a high degree of common method bias due to a cross-sectional design and data (Podsakoff et al., 2003), Harman’s one-factor was conducted. Using the condition of no factor rotation, the cumulative percentage of 41.95% was below the recommended cut off point of less than 50% (Podsakoff et al., 2003). In addition, we estimated the level of collinearity between constructs using the variance inflation factor (VIF) approach (Kock and Lynn, 2012). The highest VIF value of 1.849 obtained (Table 2) was below the cutoff point of 10 recommended by (Khan et al., 2021).

**Table 2:** Descriptive statistics, mean, standard deviation, and correlations.

Variables	1	2	3	4	5	6	7	8	9
Gender	1								
Tenure	0.153**	1							
Education	0.460**	0.053	1						
Age	0.096	0.366**	0.149**	1					
Grit	−0.011	0.033	−0.112*	−0.051	**(0.822)**				
Person organization fit	−0.028	−0.069	0.096	0.102*	0.220**	**(0.847)**			
Work engagement	0.197**	0.058	0.217**	0.128*	0.069	0.374**	**(0.889)**		
Feedback	0.215**	0.025	0.254**	0.228**	0.120*	0.311**	0.638**	**(0.848)**	
Creativity	0.088	0.030	0.199**	0.106*	0.087	0.286**	0.655**	0.621**	**(0.843)**
Mean	1.25	2.38	1.97	1.65	37.59	14.27	27.76	49.54	20.13
SD	0.436	0.875	0.717	0.842	4.551	2.803	4.975	7.313	4.457
VIF	1.342	1.214	1.362	1.259	1.102	1.275	1.836	1.849	
CMB = 41.95%									

*N* = 401; **p < 0.01 level (2-tailed); *p < 0.05 level (2-tailed). Square roots of AVEs are bracketed and bolded (constructs discriminant validity). SD, standard deviation; VIF, variance inflation factor; CMB, common method bias.

### Reliability and validity analysis

Along with a similar prior cross-sectional design technique (Xiabao et al., 2022), we conducted an exploratory and confirmatory factor analysis (EFA and CFA) of grit, work engagement, feedback, person-organization fit, and employee creativity. Governed by the rule of thumb (Hair et al., 2010, 2017), composite reliability (CR) and Cronbach’s alpha (CA) were used to calculate construct reliability for each of these constructs. Table 3 reports the indices of Cronbach’s alpha (CA) and composite reliability (CR). The indices of these two types of reliability were greater than the minimum cutoff point of 0.70 for significance, indicating that all constructs/variables in this study’s conceptual model were reliable.

**Table 3:** Constructs measurement scale and properties.

Constructs	Items	Factor loadings	CA	CR	AVE
Feedback	FQ1	0.792	0.950	0.954	0.720
	FQ2	0.857			
	FQ3	0.877			
	FQ4	0.897			
	FQ6	0.891			
	FQ7	0.905			
	FQ8	0.751			
	FQ9	0.806			
Work engagement	WE1	0.933	0.957	0.950	0.792
	WE2	0.977			
	WE3	0.879			
	WE4	0.832			
	WE5	0.818			
Creativity	EC1	0.903	0.933	0.908	0.712
	EC2	0.929			
	EC3	0.800			
	EC4	0.729			
Person organization fit	POF1	0.796	0.825	0.884	0.718
	POF2	0.831			
	POF3	0.911			
Grit	SGS1	0.730	0.720	0.926	0.677
	SGS2	0.867			
	SGS4	0.803			
	SGS5	0.702			
	SGS7	0.919			
	SGS8	0.891			

Convergent validity (Table 3) was assessed using the values of average variance extracted (AVE) for all constructs. The AVE values were greater than the minimum cutoff point (0.5) for significance. This implied that there is adequate convergent validity for all constructs (Hair et al., 2010, Hair et al., 2017). The discriminant validity of constructs was confirmed using the square root of constructs AVEs. All AVE values (Table 2) were higher than the square of their correlation coefficients. This inferred that all constructs had discriminant validity (Fornell and Larcker, 1981).

### Descriptive statistics and correlations

The means, standard deviations, and correlations of variables/constructs were calculated using the Pearson correlation approach. The Pearson correlation approach estimated the degree of straight-line relationship or strength of the linear relationship between constructs based on a correlation magnitude of 1.00 or −1.00 (Pallant, 2016). Table 2 reports the results in full detail.

### Hypothesis testing

Prior to the estimation of the hypothesis, we evaluated the fit of data to the model using the structural equation modeling technique. As shown in Table 4, the results of RMSEA, GFI, CFI, NFI, IFI, and TLI values exceeded the required values of 0.90 while chi-square statistics were less than the given cutoff point of 5.0 (Hoe, 2008). Thus, we had an acceptable fit.

**Table 4:** Model fit indexes.

Categories	Fit Indexes	Measurement	Values
Parsimonious fit	*χ*^2^/*df*	<5.00	2.609
Absolute fit	RMSEA	>0.08	0.091
	GFI	>0.90	0.903
Incremental fit	CFI	>0.90	0.952
	NFI	>0.90	0.943
	IFI	>0.90	0.925
	TLI	>0.95	0.935

RMSEA, Root Mean Square of Error Approximation; GFI, Good of Fit Index; CFI, Comparative Fit Index; NFI, Normed Fit Index; *χ*^2^/*df*, Chi-square/Degree of Freedom; IFI, Incremental Fit Index; TLI, Tucker-Lewis Index.

As illustrated in Table 5, Hypothesis 1, which states that employees’ grit has a significant positive relationship with employees’ creativity at the workplace was tested. We found a significant positive correlation for this relationship (*β* = 0.093, *p* < 0.05). This means that Hypothesis 1 was valid. Hypothesis 2 was also valid with *β* = 0.115, *p* < 0.001. This infers that employees’ grit has a significant positive relationship with employees’ work engagement at the workplace. Hypothesis 3 which claimed that employees’ work engagement has a significant positive relationship with employees’ creativity at the workplace was supported with a positive significant value of *β* = 0.649, *p* < 0.05. Employee work engagement was confirmed to mediate the relationship between employees’ grit and employees’ creativity (β = 0.781, *p* < 0.05). This validated Hypothesis 4. The final estimation validated the moderating effect of person-organization fit (*β* = 0.555, *p* < 0.05) and feedback (*β* = 0.639, *p* < 0.05). As a result, person-organization fit positively moderates the relationship between employees’ grit and employees’ work engagement (Hypothesis 5). In addition, feedback positively moderates the relationship between employees’ work engagement and employees’ creativity (Hypothesis 6). Table 5 reports the results of the path analysis.

**Table 5:** Summary of path analysis/hypotheses results

Hypothesis	Path	Coefficient (*β*)	Interpretation
H1	G->>C	0.093**	Supported
H2	G->>WE	0.115***	Supported
H3	WE->>C	0.649***	Supported
H4	G->>WE->>C	0.781**	Supported
H5	POF, G->>WE	0.555***	Supported
H6	F,WE->>C	0.639**	Supported

G, grit; C, creativity; WE, work engagement; POF, Person organization fit; F, feedback.

Furthermore, the mediating effect of work engagement in the relationship between grit and creativity was further tested following step-by-step instructions recommended by Zhao et al. (2010). Zhao et al. (2010)’s method eliminates the necessity for an initial test of the significance of the X−Y variables. Accordingly, to assess the mediating effect, the estimation of the effects of *a* = X (independent variable) on M (mediator variable) and *b* = M on the Y (dependent variable) is the only requirement. Table 6 summarizes the significant results of the mediation test for Hypothesis 4.

**Table 6:** Mediation analysis.

Path	Estimate	95% confidence interval		Result
		Lower level	Upper level	
G → WE → C	0.0320	0.0205	0.0708	Supported

G, grit; WE, work engagement; C, creativity.

## Discussion and conclusion

This study examined the relationship between grit and employee creativity through the mediating role of employee engagement and the moderating role of person-organization fit and feedback. A research model was also proposed to provide insights into the relationship between the variables mentioned earlier in this study (grit, feedback, person-organization fit, creativity). This research focus was triggered by prior emphases on grit and other organization outcome variables (Jordan et al., 2019a; Kim and Lee, 2022) and the limited research on the role of grit in performance (i.e., grit and employee creativity; Crede, 2018). The study confirmed that grit is associated with creativity as well as grit positive relationship with work engagement.

**Figure 2:**
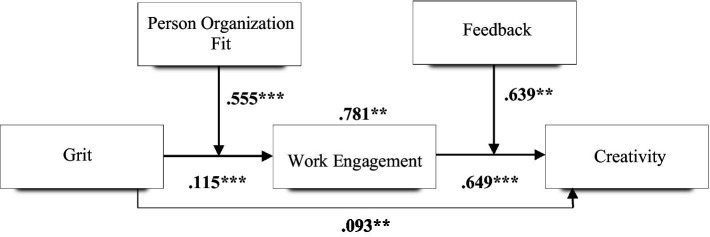
Structural model with estimates.

In general, the results support the proposed research model (Figure 1), which shows employee engagement as a positive mediator in the interplay between grit and employee creativity. The result of the mediating role of employee engagement further reaffirms its mediating role in different contexts (Salanova and Schaufeli, 2008; Sulea et al., 2012; Yalabik et al., 2013; Gupta and Shaheen, 2017; Chaudhary and Akhouri, 2018; Coetzee and van Dyk, 2018). In addition, the mediating role which employee engagement plays in the relationship between grit and employee creativity provides a more comprehensive approach to examining grit and employee creativity within an organization. Furthermore, positive relationships between grit, employee engagement, and employee creativity were also established in the results. We also found how two moderators (i.e., POF and Feedback) can strengthen the grit relationship with work engagement while feedback strengthens the relationship between work engagement and employee creativity. The results also show that person-organization fit significantly moderates the relationship between grit and work engagement, while feedback also moderates the relationship between work engagement and employee creativity. These results also reaffirm the important role that person-organization fit (Tepeci and Bartlett, 2002) and feedback (Maurer et al., 2003; Ericsson, 2009; Linderbaum and Levy, 2010; Salas et al., 2010) play within an organization. In other words, grit becomes effective in influencing employee work engagement when the person-organization fit is high. In addition, just like the indispensable moderating role of person-organization fit, feedback also plays a significant moderating role. The moderation analysis shows that work engagement is associated employee creativity when feedback is high. This study has several implications in facilitating gritty employees to remain engaged in organizational activities and request feedback on their job. This study revealed that the appropriateness of an individual work environment is most likely for a gritty person to become highly engaged with creative work activities. POF will strongly interact with grit, work engagement, and creativity especially when the organization correlates with one’s personality and the appropriate feedback is received, the organization is expected to show a strong relationship between grit and employee creativity (Figure 2).

Precisely, grit will affect creativity *via* the mediating effect of work engagement. It contributes to the literature by proposing the effect of personality variables in the workplace and situational mechanisms, addressing organizational performance results. Second, this study focused on the impact of grit on creativity, which has not been discussed much by previous researchers. Third, we add to the literature on organizational behavior by showing that grit, work engagement, person-organization fit, and feedback are all determinants of employee creativity.

### Theoretical and empirical contributions

This study contributes to TAT (Tett and Burnett, 2003) in two ways. First, we accentuate grit, work engagement, person-organization fit, and feedback as important determinants of employee creativity. Accordingly, this study extends the inadequate research on the effect of TAT’s emphasis on individuals’ traits in the workplace (Tandler et al., 2020). Specifically, we identify grit as a vital trait that influences workplace factors such as work engagement, person-organization fit, feedback, and creativity. To the best of our knowledge, no existing study has assessed the effects of these determinants of employee creativity in a work setting.

Second, our examination of the mediating and moderating effects of person-organization fit and feedback advances the TAT by providing a new understanding of the mechanisms *via* which an employee’s grit influences his or her creativity. In general, extant studies provided insight into individuals’ traits in the workplace (Duckworth and Gross, 2014; Tandler et al., 2020), yet there are no insights regarding grit and the mechanisms *via* which it contributes to employee creativity.

From the empirical standpoint, the roles of these determinants of employee creativity have received no attention in China, particularly how researchers’ grit influences their creativity. Consequently, our empirical findings provide Chinese universities with a strategic posture to increase the outcomes of researchers. Empirically, we provide evidence that can be replicated in other contexts.

### Limitations and suggestions for future research

A few limitations were identified in this study despite the many significant findings generated. The first limitation has to do with the study’s sample. The sample consisted of research assistants studying at top-notch Universities in China. This group of people cannot represent the totality of professional workers because they are only selected according to their educational level, and they cannot be compared with professional workers from other organizations. Despite these limitations, the study is the first to examine the interplay between grit, person-organization fit, work engagement, feedback, and employee creativity. This study adds to the evidence for the direct effects of grit and work engagement on employee creativity. In furtherance of this, it would be suggested that future research should consider widening the scope of the sample to cover professional workers from different organizations especially those who are not studying but are full-time workers. This will help in generalizing the research findings.

Another limitation of this study is the use of a cross-sectional research method and data, which prevents significant causal inferences or prediction of the hypothesized correlations. Although, this is a shortcoming of single response cross-sectional research (Huang, 2016), future studies can adopt other approaches such as an experimental or a longitudinal research design to examine the causal relationships between grit, employee engagement, employee creativity, person-organization fit, and feedback. Subsequent studies should also integrate more mediating and moderating variables in studying the relationship between grit and employee creativity in organizations.

## Data availability statement

The raw data supporting the conclusions of this article will be made available by the authors, without undue reservation.

## Ethics statement

The studies involving human participants were reviewed and approved by www.wjx.cn. The ethics committee waived the requirement of written informed consent for participation.

## Author contributions

MG handled the conceptualization of the study. All sections except the methodology were jointly written by MG and SD. SD handled the data collection. TA conducted the data analysis and wrote the methodology. MG proofread the paper. All authors contributed to the article and approved the submitted version.

## Conflict of interest

The authors declare that the research was conducted in the absence of any commercial or financial relationships that could be construed as a potential conflict of interest.

## Publisher’s note

All claims expressed in this article are solely those of the authors and do not necessarily represent those of their affiliated organizations, or those of the publisher, the editors and the reviewers. Any product that may be evaluated in this article, or claim that may be made by its manufacturer, is not guaranteed or endorsed by the publisher.
